# Quantifying Cell‐Derived Changes in Collagen Synthesis, Alignment, and Mechanics in a 3D Connective Tissue Model

**DOI:** 10.1002/advs.202103939

**Published:** 2022-02-01

**Authors:** Benjamin T. Wilks, Elisabeth B. Evans, Andrew Howes, Caitlin M. Hopkins, Morcos N. Nakhla, Geoffrey Williams, Jeffrey R. Morgan

**Affiliations:** ^1^ Center for Biomedical Engineering Brown University Providence RI 02129 USA; ^2^ Center for Alternatives to Animals in Testing Brown University Providence RI 02129 USA; ^3^ Department of Environmental Studies Haverford College Haverford PA 19041 USA; ^4^ Department of Molecular Biology Cell Biology & Biochemistry Brown University Providence RI 02129 USA; ^5^ Department of Pathology & Laboratory Medicine Brown University Providence RI 02129 USA; ^6^ Present address: Center for Engineering in Medicine & Surgery Harvard Medical School & Massachusetts General Hospital Boston MA 02114 USA

**Keywords:** 3D tissue engineering, collagen, connective tissue, extracellular matrix, fibroblast, fibrosis, mechanics, mechanophenotype, TGF‐β1

## Abstract

Dysregulation of extracellular matrix (ECM) synthesis, organization, and mechanics are hallmark features of diseases like fibrosis and cancer. However, most in vitro models fail to recapitulate the three‐dimensional (3D) multi‐scale hierarchical architecture of collagen‐rich tissues and as a result, are unable to mirror native or disease phenotypes. Herein, using primary human fibroblasts seeded into custom fabricated 3D non‐adhesive agarose molds, a novel strategy is proposed to direct the morphogenesis of engineered 3D ring‐shaped tissue constructs with tensile and histological properties that recapitulate key features of fibrous connective tissue. To characterize the shift from monodispersed cells to a highly‐aligned, collagen‐rich matrix, a multi‐modal approach integrating histology, multiphoton second‐harmonic generation, and electron microscopy is employed. Structural changes in collagen synthesis and alignment are then mapped to functional differences in tissue mechanics and total collagen content. Due to the absence of an exogenously added scaffolding material, this model enables the direct quantification of cell‐derived changes in 3D matrix synthesis, alignment, and mechanics in response to the addition or removal of relevant biomolecular perturbations. To illustrate this, the effects of nutrient composition, fetal bovine serum, rho‐kinase inhibitor, and pro‐ and anti‐fibrotic compounds on ECM synthesis, 3D collagen architecture, and mechanophenotype are quantified.

## Graphical abstract

A three‐dimensional (3D) scaffold‐free human connective tissue model is presented that facilitates the de novo synthesis of a highly‐aligned, collagen‐rich extracellular matrix (ECM). This model enables the direct quantification of cell‐derived changes in 3D ECM synthesis, alignment, and mechanics in response to biomolecular perturbations and may be useful for better understanding complex diseases of the ECM like fibrosis.

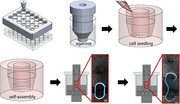

## 1 Introduction

The precise composition and spatial distribution of cells and extracellular matrix (ECM) directly correspond to tissue function. While there has been significant attention devoted to understanding how cellular dysregulation contributes to disease, more recently there has been increased focus on investigating the role that ECM dysregulation plays in disease progression. The importance of cell and matrix organization and composition is particularly apparent in connective tissues, like ligament and tendon, in which fibroblasts and collagen I are the predominant cell type and ECM component, respectively. The uniaxial alignment, high collagen density, hierarchical fibril architecture, and degree of crosslinking enables these tissues to resist high tensile loads which corresponds to their role in maintaining joint stability during skeletal motion.^[^ ^1^, ^2^ ^]^

Currently, animal models are the predominant method to study connective tissues and their associated diseases. However, the use of animals is expensive and often requires the development of transgenic models which vary in their ability to recapitulate the pathophysiology of human diseases. Moreover, although ECM proteins such as collagens are highly conserved across species, there are thousands of pathogenic gene variants for connective tissue disorders such as Marfan Syndrome, Ehlers‐Danlos syndrome and Osteogenesis Imperfecta making the development of in vivo mutation‐specific genotype‐phenotype animal studies a considerable challenge.^[^ ^3^, ^4^ ^]^ However, this could potentially be overcome by the development of more predictive in vitro models.

While there have been a number of methods developed to quantify the forces exerted by cells, there are far fewer in vitro tools to control and quantify the organization of cells, the de novo synthesis and organization of ECM, and the resulting mechanical properties in 3D.^[^ ^5^, ^6^, ^7^ ^]^ Furthermore, although there has been progress in developing complex in vitro models of ligaments and tendons,^[^ ^8^ ^]^ nearly all existing models rely on cells embedded in exogenous protein or polymer scaffolds.^[^ ^9^ ^]^ This scaffolding makes it difficult to decouple potentially confounding effects from cell‐scaffold biophysical crosstalk, which is a barrier to directly quantifying changes in cell‐mediated synthesis and alignment of de novo ECM and the resulting changes in tissue mechanics. Moreover, the majority of 2D and 3D in vitro models do not recapitulate the functional properties of connective tissues like ligaments and tendons—their mechanical properties. This is in large part because these models do not replicate the anisotropic, collagen‐dense ECM interspersed with a network of elongated fibroblast‐like cells which together are responsible for the distinct mechanical and biochemical properties of these tissues.

To address these limitations, here we describe a purely cell‐based approach to fabricating fibroblast‐derived, ring‐shaped tissue constructs that develop cell‐mediated tension, synthesize a highly‐organized human ECM de novo, and recapitulate the histological and mechanical phenotype of ligament‐like tissues. We use a multi‐scale approach integrating histology, multiphoton second harmonic generation (SHG), and electron microscopy (EM) with biochemical techniques to investigate the shift from monodispersed human primary fibroblasts to a highly‐organized 3D tissue architecture comprised of cells enmeshed in the collagen‐rich ECM they synthesized, secreted and assembled de novo. We demonstrate the role nutrient formulation and time play on the development of cellular alignment, collagen synthesis, and mechanical properties with later time points approaching physiologically relevant mechanical properties. We find that fetal bovine serum (FBS) has a dose‐dependent negative effect on the development of mechanics, which is notable considering the vast majority of in vitro cell‐based models rely on the use of serum. We demonstrate that the rho‐associated protein kinase (ROCK) inhibitor Y‐27632 has no effect on the mechanical properties of the tissues at the doses tested suggesting that ROCK signaling pathways may not be essential for the development of tensile properties. Finally, we show that Transforming growth factor (TGF)‐β1, a pleiotropic signaling molecule identified as a master mediator of fibrosis, and SB‐431542, an inhibitor of TGF‐β1, alter tissue mechanics in a dose‐dependent manner with TGF‐β1 increasing tissue stiffness and strength, a hallmark clinical feature of fibrotic disease progression, and SB‐431542 decreasing tissue stiffness and strength after 7 and 14 days of treatment.

## 2 Results

### 2.1 Structure: Characterization of tissue architecture

**Figure 1:**
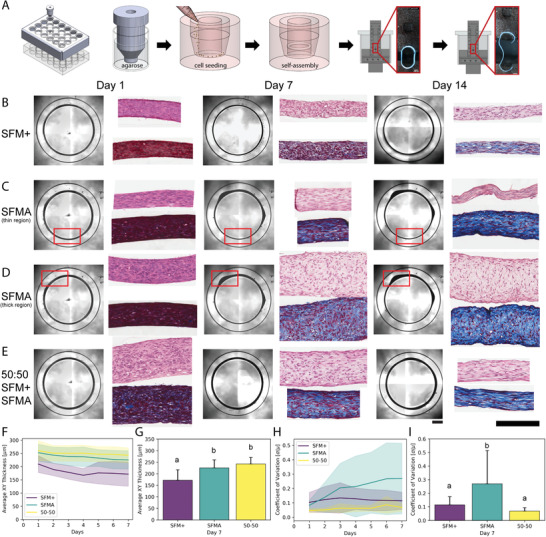
Human fibroblasts form stable 3D ring‐shaped tissues constructs whose thickness and uniformity are dependent on the composition of serum‐free media. To fabricate ring‐shaped tissues, molten agarose was added to all wells of a 24‐well plate followed by custom stainless steel inserts. After 10–15 min, the stainless steel inserts were removed and wells were left with 3D non‐adhesive agarose gels with a 5 mm diameter central inner peg surrounded by a ring‐shaped trough in which cells could be seeded, settle, self‐assemble into ring‐shaped tissue constructs, and be used for downstream assays like mechanical testing to failure at 7 to 28 day timepoints (A) After equilibrating gels for at least 24 h in serum‐free DMEM, human fibroblasts were seeded in either serum‐free media supplemented with collagen promoting supplements (SFM+), advanced DMEM (SFMA), or a 50:50 combination of the two and imaged daily (B–E) and the x‐y thickness of the tissues was measured over time (F,G). Tissues in SFM+ (B) and 50:50 (E) maintained a uniform thickness, but tissues in 50:50 were significantly thicker. Conversely, tissues in SFMA developed non‐uniform thick and thin regions as a function of time (C,D). This variation in tissue thick uniformity was quantified over 7 days (H). The average x‐y thickness (G) and the coefficient of variation of tissue thickness (I) were quantified as a function of media composition and time. Tissues in SFMA and 50:50 were significantly thicker than tissues in SFM+ at day 7 (G, One‐way ANOVA with post‐hoc Tukey HSD, *p*<0.05). The coefficient of variation of the thickness was significantly higher for SFMA compared to SFM+ and 50:50 media groups (I, One‐way ANOVA with post‐hoc Tukey HSD, *p*<0.05). Data presented as mean ± S.D., *n*=24. Scale bars = 1000, 200 µm.

To enable the fabrication of ring‐shaped tissue constructs with anisotropic tension, molten agarose was added to all wells of a 24‐well plate. Then, custom designed stainless steel inserts were simultaneously placed in each well and the agarose was allowed to solidify. After the stainless steel inserts were removed, all wells of a 24‐well plate were left with a 3D non‐adhesive gel with a 5 mm diameter central peg surrounded by a 0.75 mm cylindrical trough in which monodisperse cells could be seeded (**Figure** **1**A). Primary normal human dermal fibroblasts (NHDF) were seeded at 3 × 10^5^ cells per well in either Dulbecco's Modified Eagle's medium (DMEM) supplemented with 0.1 mm 2‐phospho‐l‐ascorbic acid and 50.0 µg mL^−1^ l‐proline (SFM+), advanced DMEM supplemented with 4 mm GlutaMax (SFMA), or a 50:50 mixture of the two (50:50) to investigate the effects of nutrient composition on tissue thickness and morphology as a function of time (Figure 1). All media groups showed a slight decrease in x‐y tissue thickness over 7 days (Figure 1F). By day 7, the average thickness of SFM+ tissue constructs was significantly smaller than both SFMA and 50:50 media groups (Figure 1G, One‐way ANOVA with post‐hoc Tukey HSD, *p*<0.05). Tissue thickness was uniform for SFM+ and 50:50 media groups, whereas tissues matured in SFMA tended to develop non‐uniform regions (Figure 1C,D). To quantify this variability in thickness, we calculated the coefficient of variation as a function of media composition and time (Figure 1H). The thickness of all media groups was relatively uniform at day 1, but by day 7 the coefficient of variation of tissues cultured in SFMA were significantly higher than SFM+ and 50:50 (Figure 1I, One‐way ANOVA with post‐hoc Tukey HSD, *p*<0.05). This suggests that media composition is directly related to the magnitude and variability of tissue thickness, with the 50:50 media formulation preserving *x*‐*y* thickness and uniformity over 7 days.

### 2.2 Histology Reveals Shift in Alignment and Proportion of Cells to ECM

To determine how changes in bulk tissue morphology were reflected in cell and ECM composition and cell alignment, we performed histology on 1, 7, and 14 day tissue constructs from each media group (Figure 1B–E). Day 1 tissues for all media groups were highly cellular and relatively disorganized with rounded cellular morphology as shown by hematoxylin and eosin (H&E) and Masson's trichrome (MT) staining. Over 14 days there was a shift in cellularity characterized by an increase in cellular alignment as well as collagen synthesis and alignment. Similar to gross tissue morphology, the shift in cell alignment and collagen synthesis and organization was media dependent. Over 14 days, collagen staining increased for SFM+ corresponding to a decrease in the proportion of cells to ECM. Masson's trichrome staining of SFMA tissues showed increased staining for collagen over time. Within a single tissue there were regional differences that also showed histological differences. Thin regions were characterized by increased cellular alignment and fibrillar collagen content, whereas thick regions had qualitative differences between the surface and bulk in which cells within the bulk were more rounded, less aligned, and the staining for collagen was more punctate (Figure 1C,D). High magnification images of thick and thin tissues corroborated region‐dependent differences (Figure S1, Supporting Information).

### 2.3 Multiphoton SHG Illuminates Changes in 3D Collagen Density

**Figure 2:**
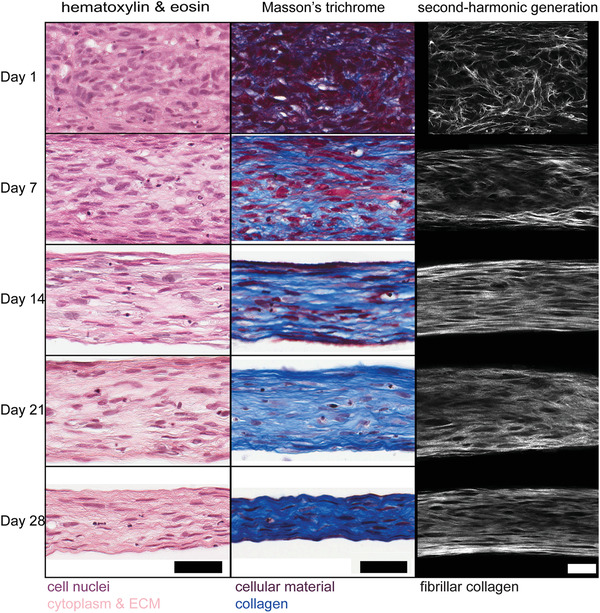
Tissue construct histology and SHG suggest an inverse relationship between cellularity and collagen content over time. Tissues cultured in 50:50 media were examined by histology (H&E and Mason's trichrome) and multiphoton second‐harmonic generation (SHG) microscopy at days 1, 7, 14, 21 and 28. In the first 14 days, there was a decrease in cellularity over time paired with an increase in cell alignment and collagen density. This shift in cellularity and cellular alignment continued through 28 days with cells increasingly elongated in the same direction as synthesized collagen. Scale bars = 100 µm.

Tissues matured in 50:50 media were characterized by a similar increase in collagen staining over time similar to SFMA, but without the development of non‐uniform thick and thin regions. To validate these histological findings and further explore the 3D architecture of fibrillar collagen, we performed multiphoton second‐harmonic generation (SHG) microscopy and compared the fibrillar collagen SHG signal to histology for tissues grown in 50:50 media over 28 days (**Figure** **2**). Isometric z‐stacks of fibrillar collagen for day 1, 14, and 28 day tissues demonstrated a significant shift in collagen architecture (Video S1, Supporting Information). Collagen was relatively sparse and disorganized at day 1, whereas z‐stacks at day 14 revealed circumferentially‐aligned fibrillar collagen. 3D reconstruction of the z‐stacks revealed an increase in 3D collagen density and organization, with the most notable differences between day 1 and day 14 (Video S2, Supporting Information). To confirm that tissues had developed cell‐mediated tension, rings matured for 28 days were carefully removed from the circular agarose wells using forceps and transferred to fixative to capture the instantaneous relaxation. SHG z‐stacks of relaxed tissues exhibited the collagen crimping behavior characteristic of collagen‐rich tensile tissues like ligaments and tendons (Video S3, Supporting Information). 3D reconstruction of collagen crimping enabled the visualization of 3D crimp topography and were compared to porcine patellar tendon (Video S4, Supporting Information).

### 2.4 Electron Microscopy Shows Nanoscale Architectural Changes

**Figure 3:**
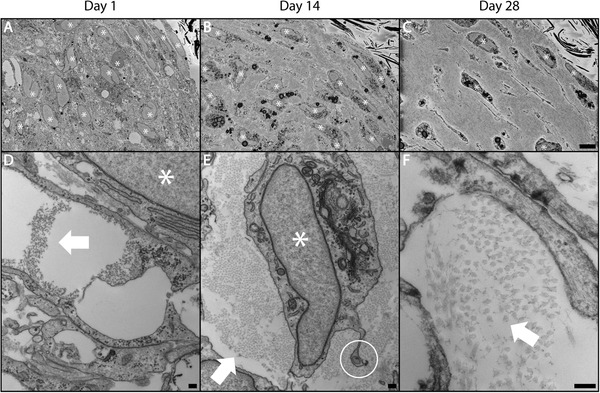
Human tissue rings secrete de novo collagen fibrils over time. Tissues cultured for 1, 14 and 28 days in 50:50 media were fixed, embedded and tissue cross‐sections were examined by serial block‐face (SBF) scanning electron microscopy (SEM) (A–C) and transmission electron microscopy (TEM) (D‐F). These images confirm a shift from purely cellular aggregates to highly‐aligned, collagen‐rich ring‐shaped tissue constructs over a period of 28 days in culture. At day 1, constructs were mostly cellular with the appearance of collagen fibrils beginning in the extracellular space (D). Day 14 revealed a shift in cellularity with individual cells clearly enmeshed in cell‐synthesized collagen and intercellular contacts maintained via long projections (E). Present are some autophagic and apoptotic cell features such as lysosomal vacuoles and nuclear degradation (A–C). By day 28, collagen fibrils appeared more separated from one another and increasing numbers of fibrils were out of plane suggesting less organization along with a continued decrease in cellularity (F). White stars indicate cell nuclei. White arrows indicate collagen fibrils. White circle highlights fibripositor. Scale bars = 500 µm (A–C), 200 nm (D–F).

While histology and SHG can visualize changes in cell and ECM composition and organization, they cannot resolve changes in individual collagen fibril synthesis and organization. To better understand the changes in cellularity and collagen synthesis at near nanoscale resolution, we performed transmission electron microscopy and serial block‐face scanning electron microscopy (**Figure** **3**). Cells appeared rounded and in direct contact with one another at 4 h in low magnification electron micrographs (data not shown). This was also supported by brightfield and SHG images of tissue constructs acquired 4 h after seeding that showed a rounded cell morphology and no fibrillar collagen (Figure S2, Supporting Information). By day 1, there was a qualitative increase in cellular organization particularly near the surface of the tissues and similarly high density of cells with the appearance of some collagen fibrils (Figure 3A,D). Day 14 tissues were characterized by a significant increase in collagen fibrils with individual cells enmeshed in newly secreted collagen and a decrease in the density of cells (Figure 3B,E). Cells maintained contacts between neighbors with long projections that could be seen interspersed between collagen fibrils. Some cells displayed indications of autophagic and apoptotic processes with the appearance of lysosomal vacuoles and nuclear degradation. Interestingly, by day 28 collagen fibrils were less packed and there were increased collagen fibrils out of plane suggesting a decrease in organization as well as a decrease in cellularity (Figure 3C,F).

### 2.5 Tissue Mechanics Corroborate Media and Time Dependent Changes

**Figure 4:**
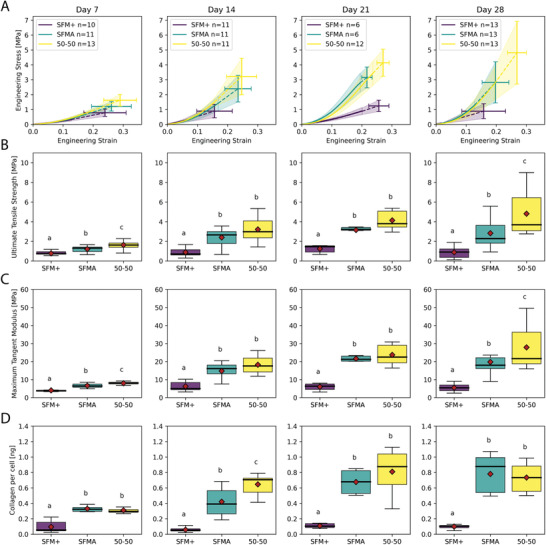
Mechanical properties and total collagen of tissue rings increase over time and is dependent on the composition of the serum‐free media. Tissue rings cultured for 7, 14, 21, and 28 days in SFM+, SFMA or 50:50 media were removed from the molds and subjected to tensile testing and analyzed for total collagen. Tissue constructs exhibited media and time dependent mechanical properties (A) that scaled with total collagen content (D). At all time points, tissue strength (B), stiffness (C), and collagen content (D) were significantly greater in 50:50 and SFMA tissues compared to SFM+ corroborating the differences visualized in tissue architecture via histology, SHG, and electron microscopy (Kruskal‐Wallis with post‐hoc Conover, *p*<0.05). While the stiffness and collagen content of 50:50 and SFMA tissue constructs were relatively consistent, by day 28, the ultimate tensile strength and maximum tangent modulus of 50:50 tissue constructs (4.81 ± 2.10, 27.8 ± 10.8 MPa) were significantly greater than SFMA (2.82 ± 1.38, 19.8 ± 6.84 MPa) and SFM+ (0.88 ± 0.50, 5.50 ± 1.91 MPa) (B‐C, Kruskal‐Wallis with post‐hoc Conover, *p*<0.05).

To quantify how changes at the cellular and molecular level affected mechanical properties, we performed mechanical testing to understand the relationship between tissue architecture, tensile properties, and collagen content. Custom grippers and a heated aqueous enclosure were fabricated to enable tensile testing of tissue constructs in 37°C phosphate‐buffered saline (PBS) on an Instron equipped with a 5N load cell (Video S5, Supporting Information). Load‐displacement curves and the cross‐sectional area of tissues were used to determine the stress‐strain relationship as a function of media composition and maturation time (Figure S3, Supporting Information). The stress‐strain curves exhibited time and media composition dependent responses that aligned with the histological changes in tissue morphology, composition, and organization (**Figure** **4**A). While the stiffness and collagen content of 50:50 and SFMA tissue constructs were relatively consistent, by day 28, the ultimate tensile strength and maximum tangent modulus of 50:50 tissue constructs (4.81 ± 2.10, 27.8 ± 10.8 MPa) were significantly greater than SFMA (2.82 ± 1.38, 19.8 ± 6.84 MPa) and SFM+ (0.88 ± 0.50, 5.50 ± 1.91 MPa) (Figure 4B,C, Kruskal‐Wallis,with post‐hoc Conover, *p*<0.05). SFM+ tissues on the other hand were significantly weaker than both SFMA and 50:50 for all time points. Similarly, SFM+ tissues also had significantly less collagen synthesis per cell compared to SFMA and 50:50 at every time point, indicating the relationship between tissue ECM composition and mechanical properties (Figure 4B–D, Kruskal‐Wallis with post‐hoc Conover, *p*<0.05). Although many groups report engineering stress‐strain equations for biomechanics studies, these equations are only valid for small deformations which is often not the case for soft tissues. Similarly, SFMA tissues exhibited high levels of variability in the cross‐sectional area which may affect mechanical calculations. To address this concern, we quantified true stress‐strain as well as performed all quantification using the minimum cross‐sectional area as well as the mean cross‐sectional area (Figures S4 and S5, Supporting Information).

### 2.6 Mechanics Depend on Initial Cell Number and Maturation Time

**Figure 5:**
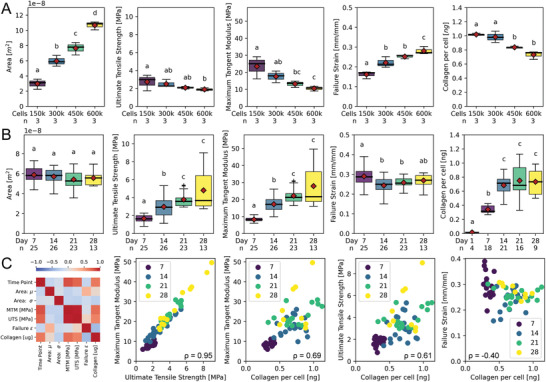
Mechanical properties of tissue rings are dependent on the number of cells seeded and maturation time. Tissue rings seeded with 1.5, 3.0, 4.5 or 6.0 x 10^5^ cells were cultured for 7, 14, 21, and 28 days in 50:50 media were measured for cross sectional area, collagen content per cell and three mechanical properties: ultimate tensile strength, maximum tangent modulus, and failure strain. Increasing the number of cells significantly increased the cross‐sectional area of the tissues (Kruskal‐Wallis with post‐hoc Conover, *p*<0.05). However, as cell number was increased, ultimate tensile strength, maximum tangent modulus, and collagen synthesis per cell all decreased, whereas failure strain increased by day 28 (A, Kruskal‐Wallis with post‐hoc Conover, *p*<0.05). A close examination of the time course of tissues formed with 3.0 x 10^5^ cells showed no significant change in cross‐sectional area or failure strain over 7 to 28 days (B). However, there was a significant increase in ultimate tensile strength and stiffness between 7, 14, and 21 day tissues corresponding to a significant increase in collagen content from 1 to 14 days (Kruskal‐Wallis with post‐hoc Conover, *p*<0.05). Spearman correlation matrix revealed strong correlations between tissue mechanics and collagen content as a function of maturation time (C). Stiffness and strength were highly correlated with ρ = 0.95. Similarly, stiffness, strength, and failure strain were significantly correlated with collagen content with ρ = 0.69, ρ = 0.61, and ρ = ‐0.40, and corresponding P‐values of 6.79 × 10^−9^, 2.49 × 10^−6^, and 0.019, respectively (Spearman Correlation with post‐hoc Bonferonni, *p*<0.05).

We further investigated the relationship between cell seeding number and maturation time for the 50:50 media group (**Figure** **5**). As expected, the tissue cross‐sectional area increased with cell seeding density from 1.5–6.0 x 10^5^ cells (Kruskal‐Wallis with post‐hoc Conover, *p*<0.05). Stiffness, strength, and collagen synthesis per cell had a significant inverse relationship with cell seeding density (Figure 5A, Kruskal‐Wallis with post‐hoc Conover, *p*<0.05). These findings may be explained by our prior work which showed that the alignment of collagen and cells was inversely related to cell seeding density, with lower seeding density corresponding to higher alignment.^[^ ^10^ ^]^ While there 50:50 media group seeded at 3 x 10^5^ cells had no significant changes in cross‐sectional area from 7 to 28 days (Figure 5B, Kruskal‐Wallis with post‐hoc Conover, *p*<0.05), there was a significant increase in tissue strength and stiffness over the first 21 days for all time points, but no significant difference between day 21 and 28 (Figure 5B, Kruskal‐Wallis with post‐hoc Conover, *p*<0.05). Interestingly, there was a concomitant increase in collagen content from day 1 to 14, but no significant changes between day 14, 21, or 28 suggesting that the majority of newly synthesized collagen occurs within the first 14 days and corresponds to an increase in tissue mechanics (Figure 5B, Kruskal‐Wallis with post‐hoc Conover, *p*<0.05). We investigated the correlation between time, cross‐sectional area, mechanical properties, and collagen content using a Spearman's rank correlation matrix (Figure 5C). Tissue strength and stiffness were highly correlated (ρ = 0.95) as were the changes in tissue strength, stiffness, and failure strain as a function of collagen content (ρ = 0.69, ρ = 0.61, ρ = −0.40, Spearman Correlation with post‐hoc Bonferonni, *p* = 6.79 x 10^−9^, 2.49 x 10^−6^, and 0.019, respectively). By color coding each time point, it was apparent that the correlation between tissue strength and stiffness versus collagen content was particularly strong for the first 14 to 21 days and began to diverge at later time points which may be a consequence of changes in collagen crosslinking or the collagen quantification method only detecting newly synthesized pepsin‐acid soluble collagen and not the transition to mature insoluble collagen that may occur at later time points.

### 2.7 Fetal Bovine Serum Negatively Regulates Tissue Mechanics

**Figure 6:**
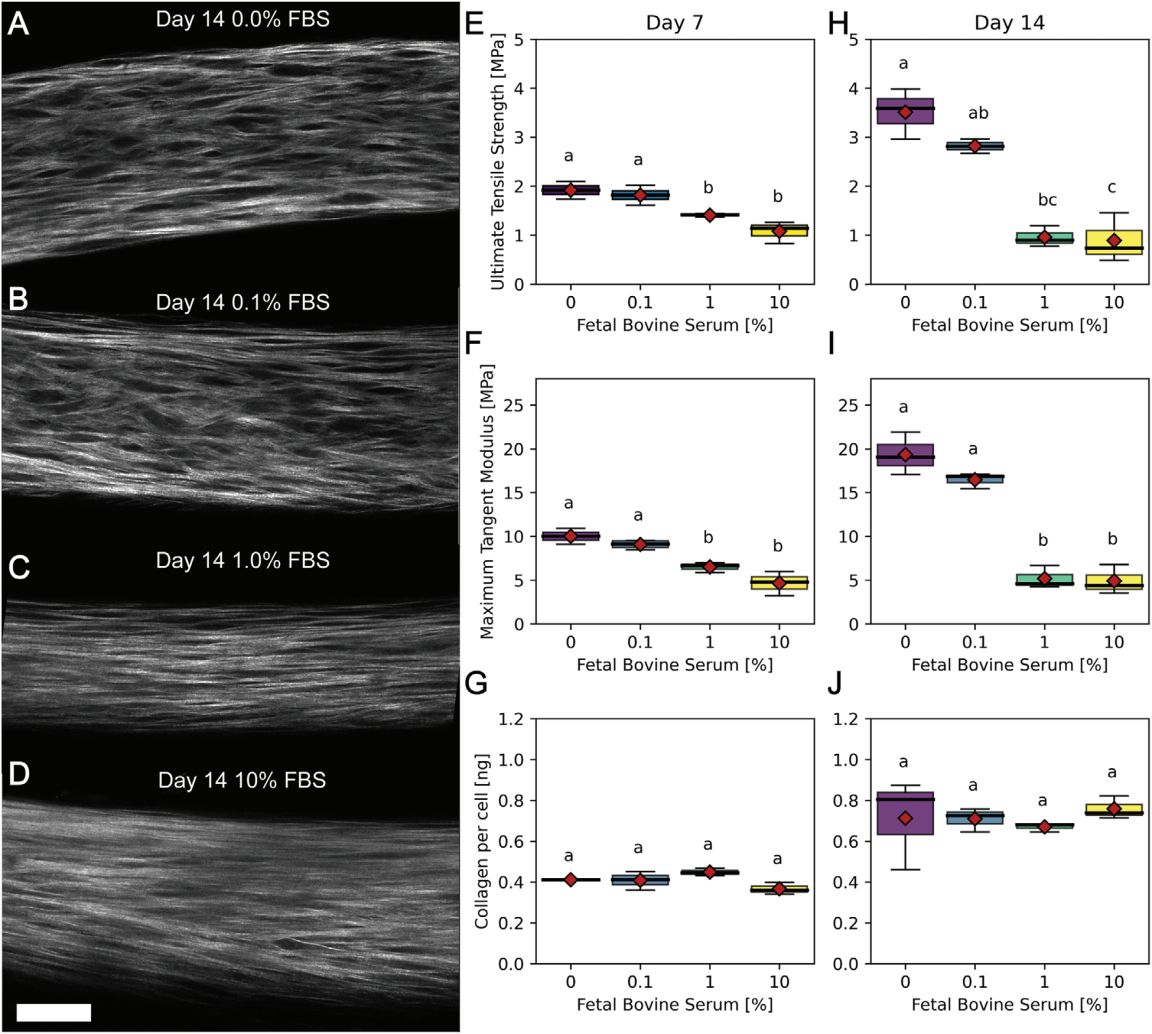
Fetal bovine serum (FBS) decreases strength and stiffness of tissues, but not total collagen content. Tissue rings seeded with 3 x 10^5^ cells that were cultured in 50:50 media with varying levels of FBS (0.0 (A), 0.1 (B), 1.0 (C) or 10% (D)) were imaged by SHG at day 14 (A–D) and measured for ultimate tensile strength, maximum tangent modulus and collagen per cell at days 7 and 14 (E–F). The SHG images of fibrillar collagen revealed qualitative differences in collagen architecture characterized by increased interfibrillar spacing in the no serum control compared to a more diffuse signal without visible dark space between fibrillar collagen in samples with serum. This shift in tissue architecture was reflected in the tissue mechanics. Ultimate tensile strength and maximum tangent modulus were highest in the no serum control at both time points. While day 14 untreated tissues had a stiffness of 19.3 ± 1.99 MPa, tissues cultured with 10% serum had a stiffness of 4.91 ± 1.37 MPa and as little as 1.0% FBS significantly decreased tissue stiffness and strength (Kruskal‐Wallis with post‐hoc Conover, *p* = 0.0045). However, there were no significant changes in collagen content at any FBS dose suggesting alternate mechanisms to changes in tissue mechanics such as crosslinking. Scale bar = 50 µm.

To better understand the response to various biological perturbations, we allowed tissue constructs to self‐assemble for 24 h and subsequently replaced media with various doses of relevant biological agents or drug compounds each time tissues were fed. While serum is a common additive in cell culture, in previous studies from our group, tissue constructs cultured with serum displayed unstable morphology characterized by the development of non‐uniform thickness, necking, and ultimately breakage of the tissue.^[^ ^10^ ^]^ To understand how the prevalent media supplement impacted the development of tissue mechanics and collagen content in this study, we supplemented 50:50 media with 10%, 1.0%, 0.1%, and 0% [v/v] FBS (**Figure** **6**). Interestingly, day 7 and day 14 treated tissues showed a significant dose‐dependent negative effect on tissue stiffness and strength compared to untreated control (Figure 6E‐J, Kruskal‐Wallis with post‐hoc Conover, *p*<0.05). For example, while day 14 untreated tissues had a stiffness of 19.3 ± 1.99 MPa, tissues cultured with 10% serum only had a stiffness of 4.91 ± 1.37 MPa Figure 6H,I. However, FBS had no effect on the total collagen synthesis at any dose or time‐point (Figure 6G,J, Kruskal‐Wallis with post‐hoc Conover, *p*>0.05). This suggests that components in FBS may be acting on the cells and, or directly on the ECM in ways that reduce the tissue strength and stiffness compared to serum‐free controls.

### 2.8 ROCK‐Inhibitor Y‐27632 Has No Effect on Tissue Mechanics

To investigate the cellular mechanisms responsible for tissue mechanics, we treated tissues with varying doses of Y‐27632, a rho‐kinase inhibitor, and tested their mechanical properties (Figure S6, Supporting Information). Interestingly, Y‐27632 did not significantly affect tissue stiffness, strength, or collagen content at 7 or 14 days (Kruskal‐Wallis with post‐hoc Conover, *p*>0.05). This suggests that the development of tissue stiffness and strength was not mediated by rho‐associated protein kinase signaling at the doses tested.

### 2.9 TGF‐β1 is Critical for Tissue Stiffness and Strength

**Figure 7:**
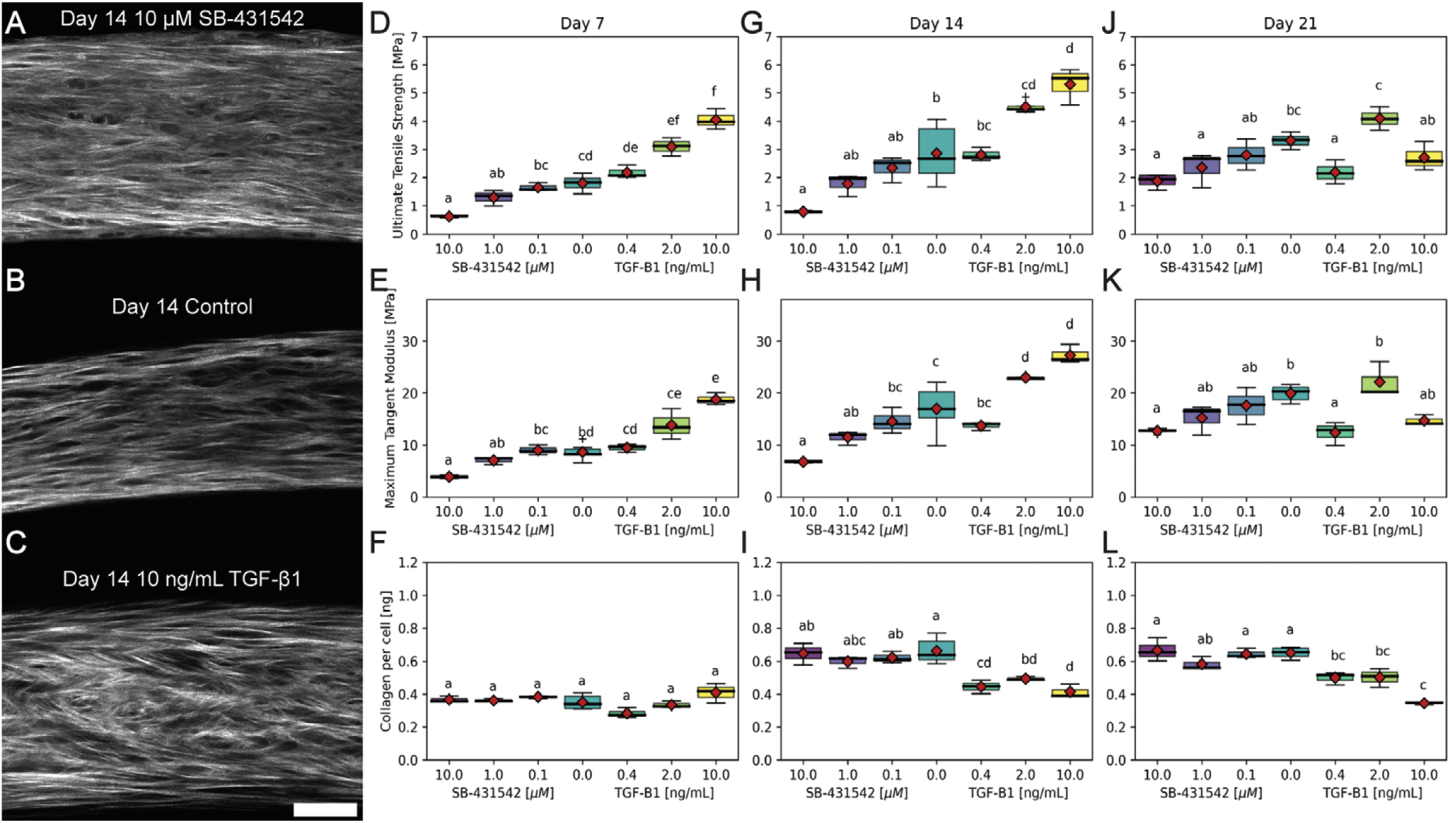
TGF‐β1 increases, whereas SB‐431542 decreases tissue mechanics. Tissue rings seeded with 3 x 10^5^ cells that were cultured in 50:50 media with varying levels of SB‐431542 (0.1, 1.0, 10.0 (A) µm), control (B), or TGF‐β1 (0.4, 2.0 or 10 ng mL^−1^ (C)) were measured for ultimate tensile strength , maximum tangent modulus, and collagen per ring at days 7 (D), 14 (E), and 21 (F). TGF‐β1 increased tissue strength and stiffness as a function of concentration after 7 and 14 days, whereas the TGF‐β1 inhibitor SB‐431542 resulted in a decrease in stiffness and strength compared to control at all time points. By day 21, the dose‐response curve abruptly shifted with a large decrease in strength and stiffness for rings treated with the highest concentration, 10 ng mL^−1^, of TGF‐β1. The highest concentration of SB‐431542, 10 µm, had the largest negative effect on tissue mechanics. However, drug treatment did not completely abrogate the development of construct strength and stiffness suggesting that there was either incomplete inhibition of TGF‐β1 signaling or TGF‐β1 independent pathways contributed to the development of mechanical properties. Interestingly, SB‐431542 had no effect on the total pepsin‐acid soluble collagen content of ring constructs at any dose or time point. There was a slight decrease in pepsin‐acid soluble collagen content of TGF‐β1 treated tissues at later time points. This may be explained by increased covalently crosslinked insoluble collagen. Scale bar = 50 µm.

TGF‐β1, a pleiotropic growth factor and proposed master mediator of fibrotic disorders, plays a critical role in ECM synthesis, remodeling, and crosslinking in health and disease. To investigate the role TGF‐β1 played on the development of tissue stiffness, strength, and collagen content, we treated tissues grown in 50:50 media with 10, 2, 0.4 ng mL^−1^ and vehicle control as well as 10, 1.0, 0.1 µm, and vehicle control SB‐431542, an inhibitor of activin receptor‐like kinase receptors (ALK) 4, 5, and 7 that blocks TGF‐β type I receptor and subsequent Smad 2/3 activation (**Figure** **7**). At day 7 and 14, the highest inhibitory concentration of SB‐431542 resulted in tissues with the weakest stiffness and strength, whereas the highest TGF‐β1 concentration increased tissue mechanics in a significant dose‐dependent manner (Figure 7D,E,G,H, Kruskal‐Wallis with post‐hoc Conover, *p*<0.05). Interestingly, there was not a concomitant change in collagen content. SB‐431542 had no effect on collagen content at any dose or time point (Figure 7F,I,L, Kruskal‐Wallis with post‐hoc Conover, *p*>0.05). However, this may be due to the collagen assay detecting newly synthesized pepsin‐acid soluble collagen fraction, whereas TGF‐β1 treatment may have an effect on collagen crosslinking and maturation leading to increased shift from soluble to insoluble collagen that may not be detected. This explanation is supported by the inability for pepsin‐acid digest to fully solubilize TGF‐β1 treated tissues, whereas control tissues were fully solubilized with the digestion protocol (Figure S7, Supporting Information). Interestingly, while there were not clearly apparent differences in SHG images for day 21 (Figure S8, Supporting Information), the shift in mechanical properties of TGF‐β1 treated tissues to highly non‐monotonic suggests the need for further characterization of cell and ECM changes for culture conditions longer than 14 days.

## 3 Discussion

The importance of ECM composition and organization in tissue homeostasis and dysregulation are becoming increasingly recognized. Consequently, there is a significant need for the development of 3D tissue models that can recapitulate native ECM architecture and function to better understand disease pathophysiology and inform the development of potential therapeutics. Unlike other models that rely on externally applied forces to direct cell alignment, to replicate ligament and tendon architecture in our model, we use a bottom‐up cell‐based tissue engineering approach to direct cellular alignment by harnessing the tendency for anchorage‐dependent cells placed on a non‐adhesive substrate to adhere to one another, self‐aggregate, and compact into a spheroid geometry.^[^ ^11^, ^12^, ^13^ ^]^ By placing a non‐adhesive peg in the center of the well, the peg acts as an inert barrier that guides the formation of a ring‐shaped tissue morphology as monodispersed cells adhere to one another, exert cytoskeleton‐mediated tension, and develop circumferential cellular alignment around the peg.^[^ ^14^, ^15^, ^16^, ^17^ ^]^ Manning et al. showed that coaction between cell adhesion molecules and cortical tension in zebrafish ectoderm aggregates led to the development of tissue surface tension corresponding to a drastic increase in cell area at the surface compared to the bulk.^[^ ^18^ ^]^ This may explain the differences we see in surface versus bulk cell and collagen organization in the histology data (Figure S1, Supporting Information). It also suggests that thinner tissues, whether from reduced initial cell seeding number or variations in regional thickness, would have increased surface tension and subsequent cell and ECM alignment due to an increase in the surface area to volume ratio, in agreement with our data. However, differences in cell and ECM alignment as a function of cell seeding density could also be explained by other phenomena like nutrient availability arising from 3D concentration gradients.

In models of tendon embryology, others have shown that actomyosin contraction is important for cellular reorganization and the development of tissue mechanics by aligning newly synthesized collagen, and that disruption of the actomyosin network with cytochalasin or blebbistatin halts the development of mechanical properties.^[^ ^19^, ^20^ ^]^ Interestingly, in our model, inhibition of rho‐kinase after 24 h of self‐assembly, which plays important roles in cellular motility, shape, and contractility, did not have a significant effect on mechanical properties or collagen synthesis over 7 or 14 days for any of the Y‐27632 doses tested (Figure S6, Supporting Information). This suggests that the development of tissue mechanics in our model can occur independently of rho‐kinase, though future studies will investigate other mediators of actomyosin contractility and more potent ROCK inhibitors.

In addition to using this model to test the role of rho‐kinase, we were also interested in characterizing the effects of growth media on ECM synthesis and mechanics due to the important role of nutrient formulation on cell signaling and phenotype.^[^ ^21^, ^22^ ^]^ Advanced DMEM (SFMA) was designed to reduce the need for serum in cell culture and contains cell survival signals like insulin, transferrin, selenium, ethanolamine, and glutathione, whereas high glucose DMEM was supplemented with a stable form of ascorbic acid (SFM+), an essential cofactor in collagen biosynthesis. Interestingly, the similarity in mechanical properties between 50:50 and SFMA tissues may suggest a minimum concentration of some component in SFMA that confers positive effects on the synthesis of collagen and development of mechanical properties, that in excess may have a deleterious effect on the uniformity in tissue cross‐section. In future studies, it would be interesting to explore other ratios of SFM+ to SFMA.

Unlike other cell types in which a quiescent state is often characterized by a decrease in metabolic activity, fibroblast quiescence is reported to have high metabolic activity with a shift in energy allocation from proliferation to cellular processes related to ECM synthesis and remodeling as well as increased macromolecule recycling via autophagy.^[^ ^23^ ^]^ Moreover, fibroblasts in vitro have been shown to remain viable in a serum‐starved state for at least 30 days prior to re‐entering the cell cycle.^[^ ^24^ ^]^ Mitogen withdrawal, contact inhibition, and loss of adhesion, each of which independently induce fibroblast quiescence, are all signals that are present in our purely cell‐based 3D model.^[^ ^25^, ^26^ ^]^ Taken together, these experimental conditions could potentially induce a shift in fibroblast phenotype from proliferative towards quiescent which may account for the rapid synthesis of ECM visualized with histology, SHG, and electron microscopy and the subsequent increase in tissue stiffness and strength in the tissue constructs. However, further characterizations are needed to investigate cell cycle and alpha‐smooth muscle actin expression to accurately assess fibroblast phenotype in our system, which unlike most systems has no exogenously added scaffold material and as a result, a distinct microenvironment. Additionally, while human dermal fibroblasts were selected for their broad accessibility and prior studies showing similar proliferation, ECM synthesis, and mechanical properties of engineered tissue constructs derived from tenocytes, in future studies it would be interesting to compare tissue specific fibroblasts, tenocytes, and other mesenchymal cells to investigate how cell origin may alter tissue construct phenotype.^[^ ^27^, ^28^ ^]^ Although the shift in the proportion of ECM to cells in our model may be related to cell state switching, it is also possible that the decrease in cellularity and collagen fibril organization and arrest in collagen synthesis from day 14 to day 28 speaks to the limits of the longevity of the culture in the absence of important nutrients or mechanical signals and future studies testing longer time points and additional supplementation with growth factors, cell survival factors, and mechanical conditioning would be valuable.

Another hypothesis that may explain a change in mechanical properties of tissues without a proportional change in total collagen such as days 14 to 28 or in serum‐treated versus serum‐starved conditions, may be due to other changes in the ECM such as collagen crosslinking. This change in crosslinking is supported by reports that show cellular proliferation via serum stimulation is inversely correlated with lysyl oxidase (LOX) expression and stability, which is controlled at both the transcriptional and post‐transcriptional level.^[^ ^29^, ^30^ ^]^ Therefore, the removal of serum may result in increased lysyl oxidase expression and stability. Interestingly, pro‐fibrotic molecule TGF‐β1 also enhances LOX and LOXL expression and stability.^[^ ^31^ ^]^ Thus, crosslinking and not total collagen content may explain the 6.5 and 4.8 fold increase in tissue strength and stiffness we see as a function of 10 ng mL^−1^ TGF‐β1 stimulation versus inhibition with 10 µm SB‐431542 at day 7. Excitingly, this tissue stiffening response is a hallmark clinical diagnostic feature of fibrotic disease progression and is supported by a recent study showing that changes in the stiffness of lung explants from healthy patients versus patients with idiopathic pulmonary fibrosis were dependent on dysregulation of posttranslational collagen crosslinking, not total collagen content and that inhibition of LOX and LOXL in an in vitro model of fibrosis reduced collagen crosslinks and tissue stiffness.^[^ ^32^ ^]^ It is also possible that changes in the synthesis and modification of ECM proteins other than collagen may affect tissue mechanics and in future studies we plan to more comprehensively assess changes in matrisome proteins using proteomic mass spectrometry similar to Merl‐Pham et al.^[^ ^33^ ^]^

In addition to using this model to investigate the effects of relevant biomolecules or drugs on ECM synthesis and tissue mechanics, we believe this model could be adapted to investigate the effects of specific gene mutations on ECM synthesis, organization, and mechanics relevant to connective tissue diseases like Marfan Syndrome or Loeys‐Dietz syndrome whose pathophysiology is directly related to deregulated TGF‐β signaling and subsequent changes in tissue mechanics that result in mechanical failure but which the exact spatiotemporal regulation of TGF‐β signaling is still largely unknown.^[^ ^3^ ^]^ Moreover, the relationship between gene variant and benign versus pathogenic phenotype for many of these diseases has not been fully characterized causing difficulties with clinical diagnosis and patient treatment strategy.^[^ ^34^ ^]^ Controlling for variables like age, sex, and passage number, in the future we plan to apply conditional gene knockout and reconstitution to explore how specific mutations, such as in COL1A1 for Osteogenesis Imperfecta, or FBN1 for Marfan Syndrome, result in quantitative and qualitative changes in collagen synthesis and tissue mechanics in our model.^[^ ^35^ ^]^

Another feature of cell‐based in vitro models of ligaments and tendons is that they are generally far weaker mechanically than native tissues. Because our tissue histology showed features of connective tissue architecture like uniaxial cell alignment, collagen synthesis, and collagen crimping, we quantified how these changes in architecture were reflected in tissue mechanics due to their functional capacity to resist and transmit tensile forces and compared to in vivo tissue mechanics. While native ligament mechanics range 1‐2 orders of magnitude based on anatomical location and function, peritoneal or suspensory ligaments like the uterosacral ligament, have a stiffness ranging from 14.1 ± 1.4 MPa which is well within the range of our 50:50 media group tissue constructs by 14 days of maturation which had a stiffness of 18.2 ± 4.57 MPa.^[^ ^36^ ^]^ Although articular ligaments typically have higher mechanical properties, the dorsal intercarpal and dorsal radiocarpal of the wrist have stiffnesses reported from 29.34 ± 12.1 and 46.0 ± 16.1 MPa, respectively which, excitingly, is comparable to the strongest experimental group reported here, the day 28 50:50 tissue constructs, which had a stiffness of 27.8 ± 10.8 MPa.^[^ ^37^ ^]^ However, there are ligaments with stiffnesses on the order of 100s of MPa like the anterior cruciate ligament (ACL) of the knee^[^ ^38^ ^]^ and in future studies, it would be interesting to test additional strategies that simulate skeletal motion like mechanical conditioning to further increase tissue mechanics.^[^ ^8^ ^]^

## 4 Conclusion

There is an acute need for engineered tissue models that are predictive of human health and disease. With respect to connective tissue dysregulation and disease, in vitro models need to recapitulate the matrix‐rich anisotropic architecture of these tissues in order to mirror their mechanical phenotype which is a direct measure of their function. While there remain significant challenges towards generating purely cell‐based engineered tissues with mechanical properties and architecture that resemble mature anisotropic collagen‐rich tissues, here we presented a relatively straightforward approach that enables the direct assessment of structure‐function relationships in response to targeted biological perturbation in a lab grown connective tissue model. Although future studies are necessary to further characterize model longevity and how mechanical conditioning may affect tissue maturation, we believe this in vitro human connective tissue model may be useful to others interested in investigating the role of mechanotransduction pathways, media composition, growth factors, or drugs on cell‐mediated synthesis and alignment of ECM and how changes in 3D ECM composition and architecture alter tissue mechanics and function such as in diseases like fibrosis.

## 5 Experimental Section

### Cell Source and Culture Conditions

Juvenile normal human dermal fibroblasts (jNHDF) were purchased from PromoCell (Heidelberg, Germany). Cells were expanded in Dulbecco's Modified Eagle's medium (DMEM) with high glucose, l‐glutamine, phenol red, and sodium pyruvate (11995065, Thermo Fisher Scientific, Waltham, MA) supplemented with 10% FBS and 1% penicillin/streptomycin at 10% CO_2_ and 37 °C. Cells were passaged (P3‐8) and expanded using a standard trypsin protocol. Cells were rinsed with 1x PBS (SH30256.FS, Thomas Scientific, Swedesboro, NJ), exposed to 0.05% trypsin in PBS for 4 min, quenched with serum containing media, centrifuged at 220g for 3 min, resuspended, counted, and plated at a density of 3.5–7.0 x 10^3^ cells per cm^2^. Ring‐shaped tissue constructs (5 mm) were seeded at 3 x 10^5^ cells per well in 1 mL of media exchanged every 2/3 days unless otherwise noted. For media composition experiments, tissues were cultured in serum‐free DMEM supplemented with 0.1 mm 2‐phospho‐l‐ascorbic acid trisodium salt (Sigma‐Aldrich, St. Louis, MO), 50.0 µg mL^−1^ l‐proline (Thermo Fisher), 1% penicillin/streptomycin (SFM+), advanced DMEM (12491015) supplemented with 4 mm GlutaMax and 1% penicillin/streptomycin (SFMA), or a 50:50 mixture of the two (50:50 SFM+/SFMA).

### Fabrication of Ring‐Shaped Tissue Constructs

Ring‐shaped molds were designed using CAD software (SolidWorks Corporation, Concord, MA) and fabricated from stainless steel 316L on a CNC lathe (Protolabs, Maple Plain, MN). The stainless steel parts were designed to fit into a single well of a standard 24 well plate. Ring‐shaped agarose gels were cast by placing the stainless steel part into a well filled with 1.5 mL of molten, sterile 2% (w/v) agarose (BP160‐500, Fisher Scientific, Hampton, NH) in 1x PBS, allowed to harden for 15 min, and removed. This resulted in an agarose gel with an inner peg diameter of 5 mm surrounded by a 0.75 mm cylindrical trough. A lid mirroring a standard 24 well plate lid was fabricated out of aluminum with through holes above each well to permit the placement of all 24 stainless steel parts simultaneously. Gels were equilibrated in serum‐free DMEM with 1% penicillin/streptomycin at 37°C, 10% CO_2_ for at least 24 h before use.

### Drug Treatment

For all dose‐response experiments, ring constructs were allowed to form for 24 h prior to treatment. FBS (12483020, Life Technologies, Carlsbad, CA), Y27632 (S1049, Selleck Chemicals, Houston, TX), TGF‐β1 (7754‐BH‐25, Selleck Chemicals), and SB‐431542 (S1067, Selleck Chemicals) doses were freshly prepared from frozen aliquots and resuspended in 50:50 SFM+/SFMA medium prior to feeding 3x per week. Doses of FBS were prepared at 10%, 1.0%, and 0.1% [v/v] concentrations with a 0% control. Y‐27632 and SB‐431542 were reconstituted in dimethyl sulfoxide (DMSO) and resuspended at a final concentration of 10, 1.0, 0.1, and a 0 µm vehicle control with a DMSO final volume always less than 0.1%. TGF‐β1 was reconstituted in 0.1% bovine serum albumin (BSA) in 4 mm HCl (RB04, Selleck Chemicals) and prepared at a final concentration of 10, 2, 0.4, and 0 ng mL^−1^ vehicle control.

### Histology

Agarose gels containing fibroblast rings were transferred to a new 24 well plate, washed with PBS, fixed with 10% buffered formalin, and kept at 4 °C until ready to process. Prior to embedding, formalin was removed and fresh 2% [w/v] agarose solution was added to the top of the gels to encapsulate the tissue. After cooling, the gels containing the fixed tissues were carefully removed from their 24 well plates using a spatula and moved to cassettes that were embedded in paraffin. After processing, samples were cut into sections 5‐8 µm thick using a Leica RM2265 microtome (Leica Microsystems, Wetzlar, Germany). Sections were stained for either hematoxylin and eosin (Richard‐Allan Scientific; Thermo Scientific), or Masson's trichrome (Electron Microscopy Sciences, Hatfield, PA, USA) according to manufacturer protocols. Stained slides were digitized using an Aperio ScanScope CS (Leica Microsystems, Wetzlar, Germany).

### Collagen Quantification

Tissues were homogenized with 2 mm glass beads for 2/3 min and solubilized in 0.1 mg mL^−1^ pepsin in 0.5 m acetic acid at 4°C for 48 h. Total pepsin‐acid soluble collagen was quantified via sircol dye binding assay per kit instructions (S5000, Biocolor, United Kingdom). Absorbance was measured at 525 nm and collagen content was determined from a standard curve and adjusted to microgram equivalent of native pepsin‐acid soluble collagen.

### Mechanical Testing

Ring‐shaped microtissues were mechanically tested using custom designed grippers on a uniaxial tensile testing setup (Instron 5943, Norwood, MA) equipped with a 5N (5 mn resolution) load cell. Agarose molds were trimmed with a razor blade and top and side‐view brightfield images were obtained on a Nikon Eclipse Ts2 microscope (Nikon, Tokyo, Japan) to determine the initial elliptical cross‐sectional area (*A*_0_). Just prior to testing, tissue constructs were gently removed from the agarose mold using forceps and mounted between grippers which consisted of two horizontal cylindrical segments with a circular diameter of 3 mm fabricated from glass filled nylon (PA614‐GS, ProtoLabs). Tissues were brought to a 5mm initial grip‐to‐grip distance and tests were performed in PBS at 37 °C. Unless otherwise noted, tests were performed at a strain rate of 0.1% initial length per second, corresponding to 5 µm per second for 5 mm diameter rings to approach quasi‐static loading. Load (*N*) and extension (mm) were sampled at 20 Hz and tests automatically terminated when a drop in load greater than 40% was detected which corresponded to tissue failure. Tests were optically recorded using a custom imaging setup equipped with a BlackflyS, Color Camera (BFS‐U3‐200S6C‐C USB3, Edmund Optics, Barrington, NJ) sampled at 2 Hz.

### Mechanical Data Analysis and Visualization

Mechanical parameters were quantified with Python 3.6 (Python Software Foundation) using SciPy, NumPy, Pandas, Matplotlib, scikit‐image, scikit‐posthocs, and statsmodels modules.^[^ ^39^, ^40^, ^41^, ^42^, ^43^, ^44^, ^45^ ^]^ Raw load data were normalized to remove the drag force measured by running the test protocol in PBS at 37°C without a sample loaded on the grippers. Mechanical testing protocol was adapted from Adebayo et al. and Gwyther et al.^[^ ^46^, ^47^ ^]^ The tissue was considered in tension and the gauge length was recorded when the raw load was greater than 5 mn. Engineering stress (*N*/2*A*_0_) and engineering strain (Δ*L*/*L*_gauge_) were analyzed to determine the maximum tangent modulus (MTM), ultimate tensile strength (UTS), and failure strain, where A_0_ was the elliptical cross‐sectional area. The tangent modulus or slope of the stress–strain curve was obtained by acquiring linear fits over a region of 8% strain for all points on the curve as described in Ristaniemi et al.^[^ ^48^ ^]^ In all tests, the maximum tangent modulus (MTM) fell within the “linear region” of the stress‐strain curve. This was calculated to compare to existing biomechanical literature which often uses maximum tangent modulus, Young's Modulus, and stiffness terminology interchangeably. Engineering stress and strain are only valid for small deformations, which is not the case for many biological specimens. True stress and strain were also quantified assuming tissue volume conservation and uniform deformation. For all mechanical analyses, along with engineering stress‐strain, the true stress‐strain and analyses using minimum and mean cross‐sectional area are available in the Supporting Information.

(1) $$\mathrm{EngineeringStrain}=\Delta L/L_{\mathrm{gauge}}$$

(2) $$\mathrm{EngineeringStress}=N/(2A_{0})$$

(3) TrueStrain=ln(1+EngineeringStrain)

(4) TrueStress=EngineeringStress(1+EngineeringStrain)

### Multiphoton Second‐Harmonic Generation (SHG) Microscopy

Fibrillar collagen was visualized using an Olympus FV‐1000‐MPE multiphoton microscope (Olympus, Tokyo, Japan) equipped with a Mai Tai HP tunable laser with the excitation wavelength set to 790 nm and a 405/40 filter cube to select for fibrillar collagen second‐harmonic signal. Similar to histology processing, agarose gels containing fibroblast rings were transferred to a new 24 well plate, washed with PBS, fixed with 10% buffered formalin, and kept at 4 °C until imaging. Tissue constructs were imaged in situ without removing them from the agarose mold using a 25x (Numerical Aperture 1.05, Working Distance 2 mm) dipping objective in PBS. Collagen crimp images were obtained by gently removing tissue rings from the agarose mold using forceps and immediately placing the rings into fixative to capture the instantaneous relaxation.

### Electron Microscopy Sample Preparation

Specimens were prepared by adapting an enhanced contrast protocol.^[^ ^49^ ^]^ Specimens were fixed in 2.5% glutaraldehyde in 0.15 m sodium cacodylate, 2% paraformaldehyde, and 2 mm calcium chloride overnight or longer at 4°C then washed 5x for 3 min each in cold 0.15 m sodium cacodylate buffer with 2 mm calcium chloride. Specimens were then incubated for 1 h on ice in a solution comprised of equal volumes of 4% osmium tetroxide and 3% potassium ferrocyanide in a 0.3 m cacodylate buffer with 4 mm calcium chloride prepared immediately prior to use. Specimens were then washed 5x for 3 min each in filtered milliQ H_2_0 and then incubated at room temperature for 20 min in a fresh solution of filtered 94.2 mm thiocarbohydrazide (TCH) in milliQ H_2_0. Specimens were then washed 5x for 3 min each in filtered ddH_2_0, incubated in 2% osmium tetroxide in milliQ H_2_0 for 30 min and then washed 5x for 3 min each in filtered milliQ H_2_0. Specimens were placed in 1% uranyl acetate in ddH_2_0 overnight at 4°C and then washed 5x for 3 min each in filtered milliQ H_2_0. Specimens were then incubated in Walton's lead aspartate solution (20 mm lead nitrate, 30 mm aspartic acid solution, pH 5.5) at 60°C for 30 min and then washed 5x for 3 min in filtered milliQ H_2_0. Specimens were dehydrated in graded ethanol solutions of 20%, 50%, 70%, 95% for 20 min each followed by dehydration in 100% ethanol 3 x 20 min followed by 24 h in 100% ethanol.

For scanning electron microscopy (SEM), dehydrated samples were critical point dried with liquid CO_2_, and sputter coated with 100 Å gold–palladium and visualized with a Thermo Fischer Scientific Apreo VS scanning electron microscope. For transmission electron microscopy (TEM) and serial block‐face (SBF) scanning electron microscopy, dehydrated samples were rinsed with propylene oxide 1x for 20 min and then infiltrated with fresh propylene oxide for up to 24 h.^[^ ^49^ ^]^ Samples were embedded in Epon resin at 1:3, 1:1, 3:1, 1:0 Epon:propylene oxide on a rotator for 12 h each. Samples were then transferred to fresh 100% Epon for 6 h and cured. 80 nm sections were visualized on a Phillips EM 410 transmission electron microscope.

### Statistical Analysis

All data including error bars in bar and box plots and shaded regions in line plots are represented as the mean ± standard deviation. For box plots, the horizontal black line represents the median and the red diamond represents the mean. Homoscedasticity and normality were tested with Levene's test and Shapiro–Wilk test, respectively. To determine statistical significance between multiple groups, for parametric One‐way ANOVA with post‐hoc Tukey HSD was used or non‐parametric Kruskal‐Wallis test with post‐hoc Conover was used. Experimental groups labeled with unique letters denote statistical significance of *p* < 0.05 whereas experimental groups labeled with shared letters denote not statistically significant within a subplot. All statistics were performed using Python.

## Conflict of Interest

J.R.M. has an equity interest in Microtissues, Inc. This relationship has been reviewed and is managed by Brown University in accordance with its conflict of interest policies.

## Supporting information

Supporting Information

Supplemental Video 1

Supplemental Video 2

Supplemental Video 3

Supplemental Video 4

Supplemental Video 5

## Data Availability

The data that support the findings of this study are available from the corresponding author upon reasonable request.
