# Supplementary material for: Quantifying Cell‐Derived Changes in Collagen Synthesis, Alignment, and Mechanics in a 3D Connective Tissue Model
Source: Adv Sci (Weinh). 2022 Feb 1;9(10):2103939. doi: 10.1002/advs.202103939 (PMC8981917; doi:10.1002/advs.202103939)
Supplement: Supplementary file 1 — Supporting Information [file ADVS-9-2103939-s006.pdf]

ADVANCED SCIENCE
Open Access

Supporting Information

for *Adv. Sci.*, DOI 10.1002/advs.202103939

Quantifying Cell-Derived Changes in Collagen Synthesis, Alignment, and Mechanics in a 3D Connective Tissue Model

*Benjamin T. Wilks, Elisabeth B. Evans, Andrew Howes, Caitlin M. Hopkins, Morcos N. Nakhla, Geoffrey Williams and Jeffrey R. Morgan**

ADVANCED SCIENCE Open Access

Supporting Information

for *Adv. Sci.,* DOI: 10.1002/advs.202103939

Quantifying Cell-Derived Changes in Collagen Synthesis, Alignment, and Mechanics in a 3D Connective Tissue Model

*Benjamin T. Wilks, Elisabeth B. Evans, Andrew Howes, Caitlin M. Hopkins, Morcos N. Nakhla, Geoffrey Williams, and Jeffrey R. Morgan**

Supplemental Figures

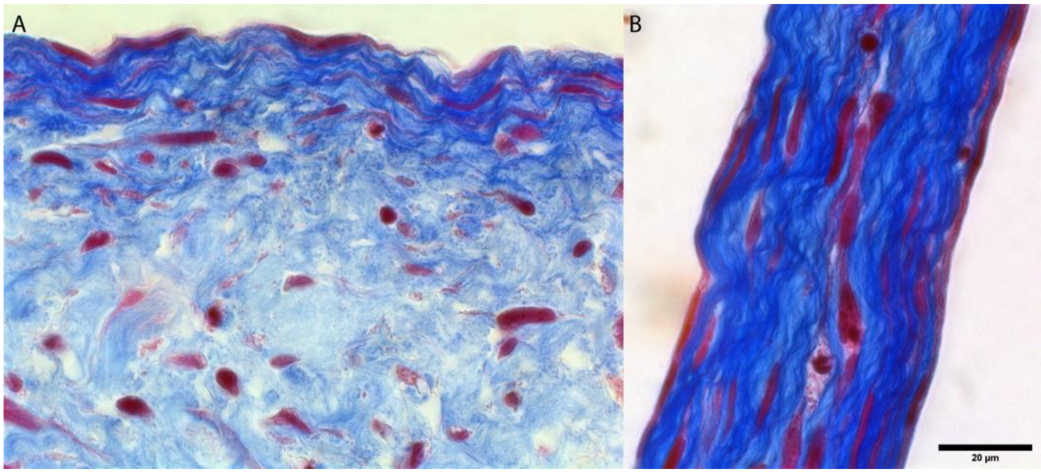

Supplemental Figure 1. Tissues have local variations in collagen and cell morphology. Tissue rings seeded with 3 x 10^5 cells that were cultured in SFMA media for 14 days were fixed, embedded and sections stained with Masson's trichrome. Shown are images from thick (A) and thin (B) regions. The outer surface of thicker regions was characterized by cellular elongation and abundant fibrillar collagen aligned in the same direction as cells. Moving inward from the surface, there was a clear transition to a more rounded cellular phenotype and punctate collagen signal in the bulk region of the tissue. In contrast, the thin regions of tissues were characterized by highly aligned cells and abundant fibrillar collagen throughout. This suggests that the gross tissue morphology was directly related to the alignment of cells and synthesis of fibrillar collagen with thinner tissue constructs that have an increased proportion of surface to bulk regions corresponding to increased alignment and collagen. Scale bar = 20 μm.

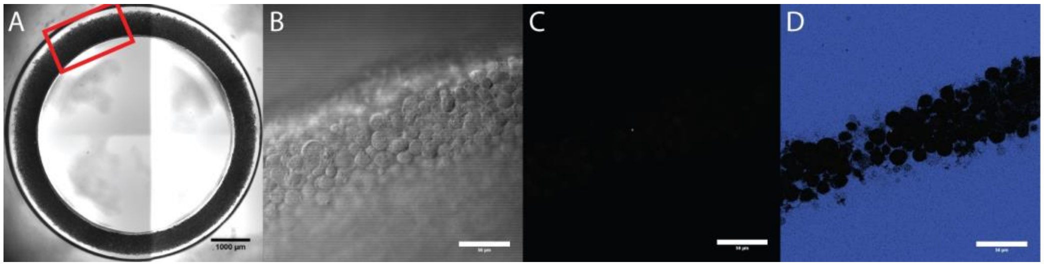

Supplemental Figure 2. Early tissue constructs have a rounded cellular morphology and little to no fibrillar collagen. Tissue rings seeded with 3 x 10^5 cells in 50:50 media self-assembled for only 4 hours prior to being fixed and imaged by transmitted light (A) low magnification, (B) high magnification and (C, D) SHG. Transmitted light images enabled the visualization of bulk tissue morphology and individual cells which were characterized by high cell density and packing and a rounded-cell morphology. Minimal fibrillar collagen was detectable in the SHG signal (C). Inversion of the SHG image via a lookup table enabled the detection of individual cell outlines. Scale bars = 1000 μm (A), 50 μm (B-D)

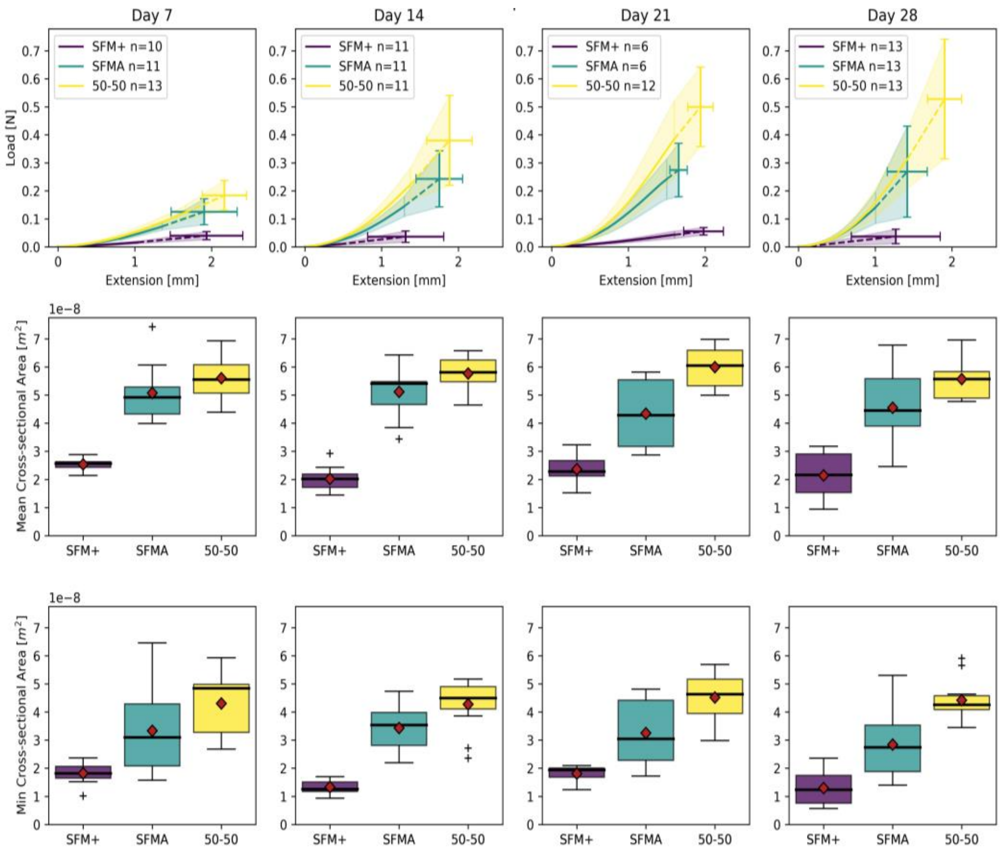

Supplemental Figure 3. Load-displacement curves and tissue cross-sectional area were used to quantify stress-strain characteristics. Tissue rings seeded with 3 x 10^5 cells were cultured in SFM+, SFMA or 50:50 media were measured for load, maximum cross-sectional area and minimum cross-sectional area at days 7, 14, 21, 28. To determine stress-strain characteristics, load was normalized by mean cross-sectional area of tissue constructs. Because the geometry of the tissue was a ring, the cross-sectional area was multiplied by a factor of two prior to normalizing the load. Some variation in stress-strain quantification may arise from regional variation in tissue cross-section.

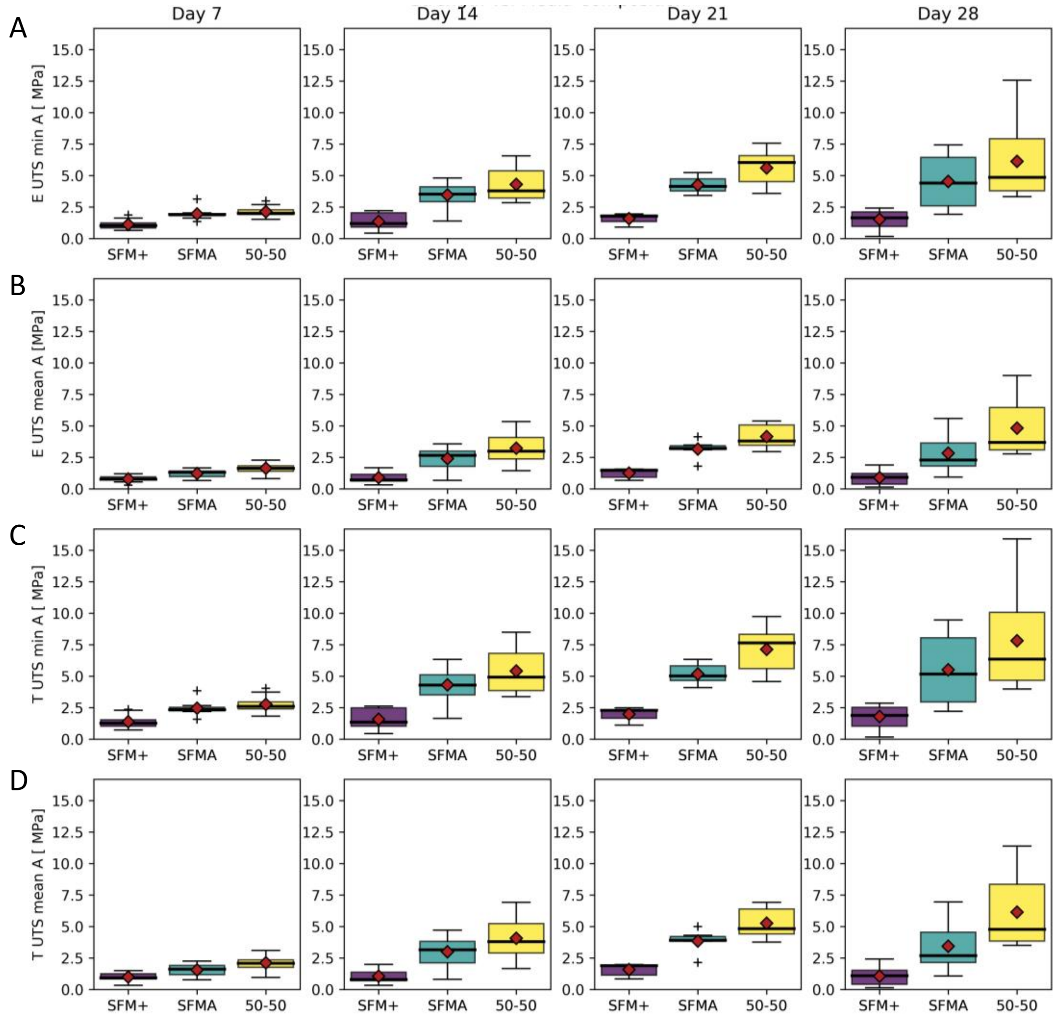

Supplemental Figure 4. Strength of tissue rings is determined by whether the engineering or true stress-strain equations are used and by how cross-sectional area is quantified. Tissue rings seeded with 3 x 10^5 cells were cultured in SFM+, SFMA or 50:50 media were measured for ultimate tensile strength (UTS). Same data were used to compute minimum and mean engineering UTS (A, B) versus minimum and mean true UTS (C, D). Engineering stress-strain curves are most commonly used in biomechanical literature enabling direct comparison to other studies. However, engineering stress and strain are only valid for small deformations, which is not the case for most soft tissue biological specimens. True stress and strain were also quantified assuming tissue volume conservation and uniform deformation, which are also imperfect assumptions. A comparison of tissue construct strength as a function of media and time when calculated using the average cross-sectional versus the minimum cross-sectional area as well as engineering versus true stress-strain equations. In future studies, the incorporation of 3D digital image correlation would enable the direct quantification of regional changes in tissue geometry throughout the test.

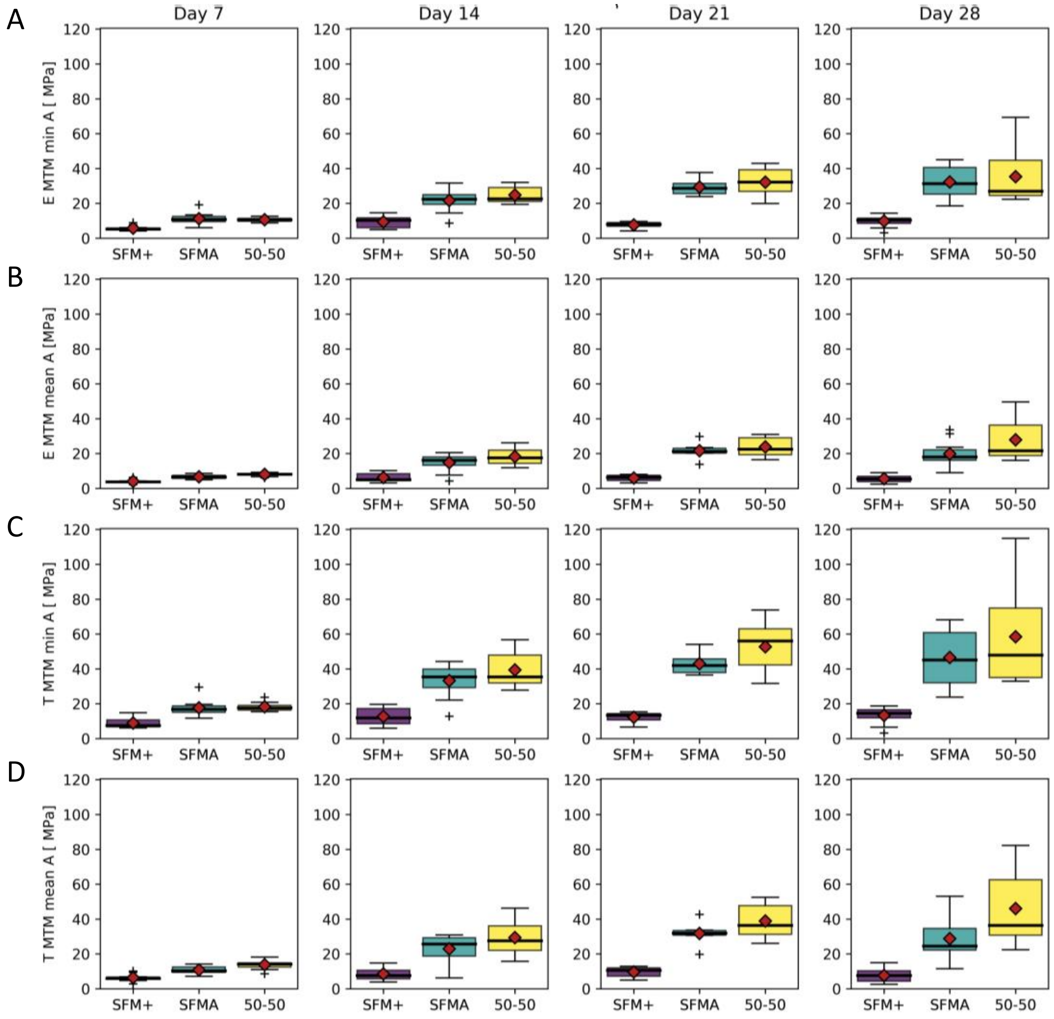

Supplemental Figure 5. Stiffness of tissue rings is determined by whether engineering or true stress-strain equations are used and by how cross-sectional area is quantified. Tissue rings seeded with 3 x 10^5 cells were cultured in SFM+, SFMA or 50:50 media were measured for maximum tangent modulus (MTM). Same data were used to compute minimum and mean engineering MTM (A, B) versus minimum and mean true MTM (C, D) using minimum and mean cross-sectional area measurements, respectively.

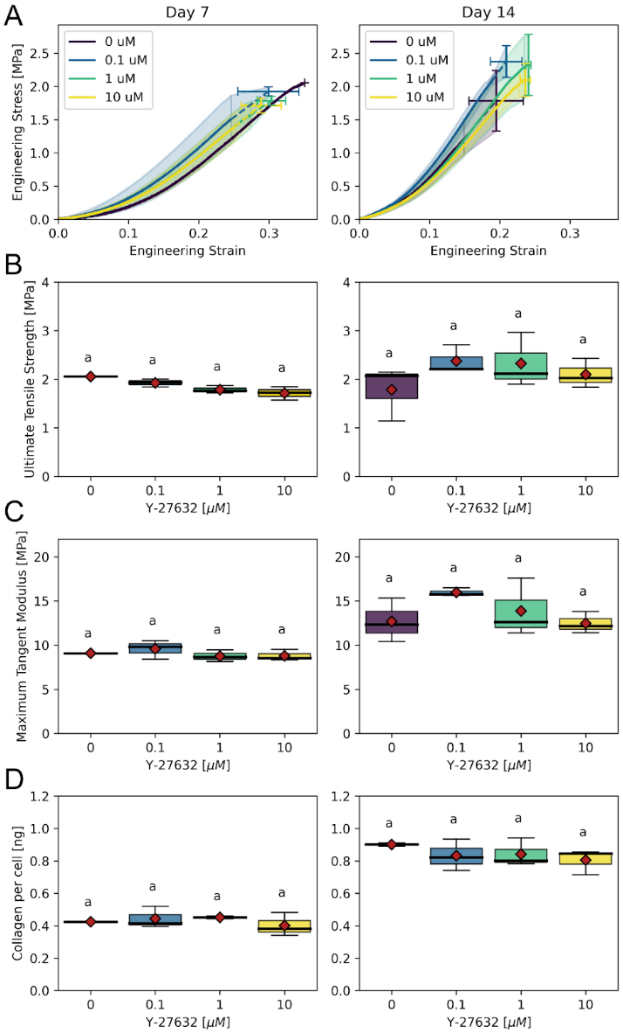

Supplemental Figure 6. Inhibition of Rho-kinase had no effect on tissue mechanics or levels of collagen. Tissue rings seeded with 3 x 10^5 cells that were cultured in 50:50 media with varying levels of Y27632 (0.1, 1.0, 10.0 μM) starting 24 hours after addition of cells were measured at days 7 and 14 for engineering stress (A), ultimate tensile strength (B), maximum tangent modulus (C) and collagen content per ring (D). Y27632, a rho kinase inhibitor, that inhibits cytoskeletal force generation might be expected to interfere with the development of cell and ECM alignment and maturation. Interestingly, there was no effect on tissue mechanics or levels of collagen at 7 or 14 days. This may be due to most of the cellular reorganization and alignment happening within the first 24 hours of self-assembly prior to the addition of the drug or the doses are not high enough to exert an effect in a 3D tissue model.

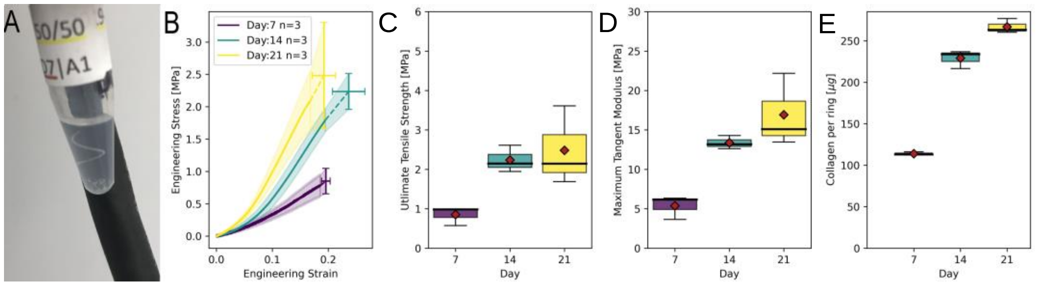

Supplemental Figure 7. Rings treated with TGF-β1 did not solubilize completely in the pepsin-acid digest compared to untreated rings suggesting a potential increase in collagen crosslinking in response to TGF-β1 (A). Tissue rings seeded with 3 x 10^5 cells that were cultured in 50:50 media with 2 ng/mL TGF-β1 and 10 μM SB-431542 were measured for engineering stress, ultimate tensile strength, and collagen per ring at days 7, 14 and 21. Combination treatment of 10 μM SB-431542 and 2 ng/mL TGF-β1 continue to develop mechanical strength and stiffness with time over 21 days however the increase is significantly reduced compared to control (B-D). Interestingly, the combination SB-431542 and TGF-β1 treatment had similar pepsin-acid collagen quantities as compared to control supporting the hypothesis that crosslinking mediated changes in mechanical properties, not the total amount of collagen (E).

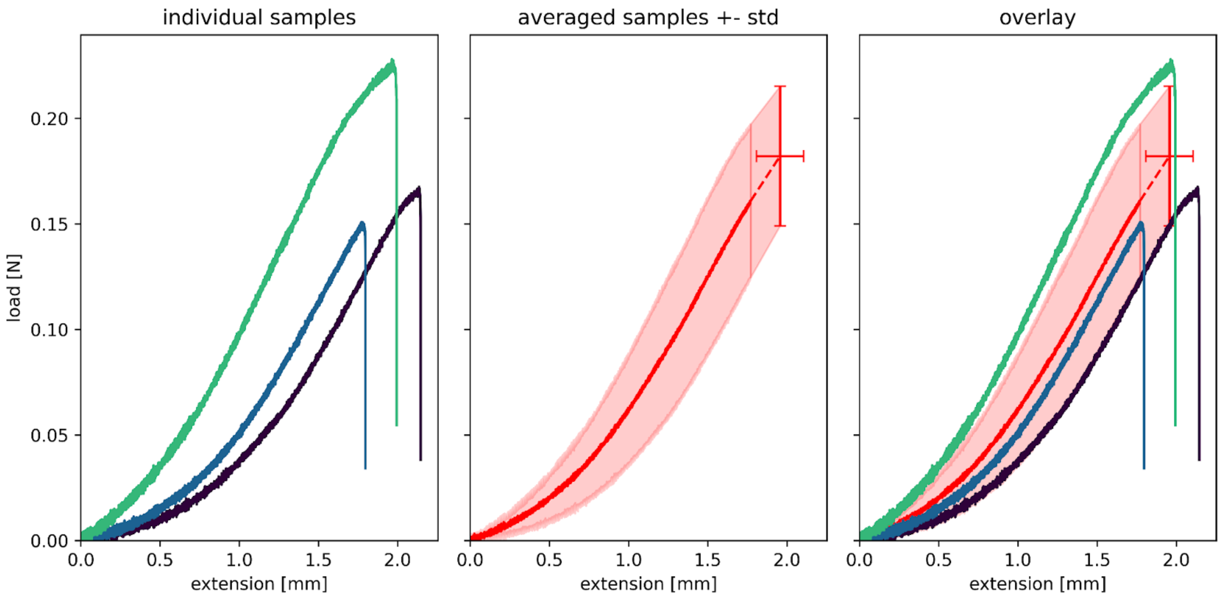

Supplemental Figure 8. Mechanical stress-strain data was averaged with shaded regions and error bars representing standard deviation to show the variability in data including failure stress and strain.

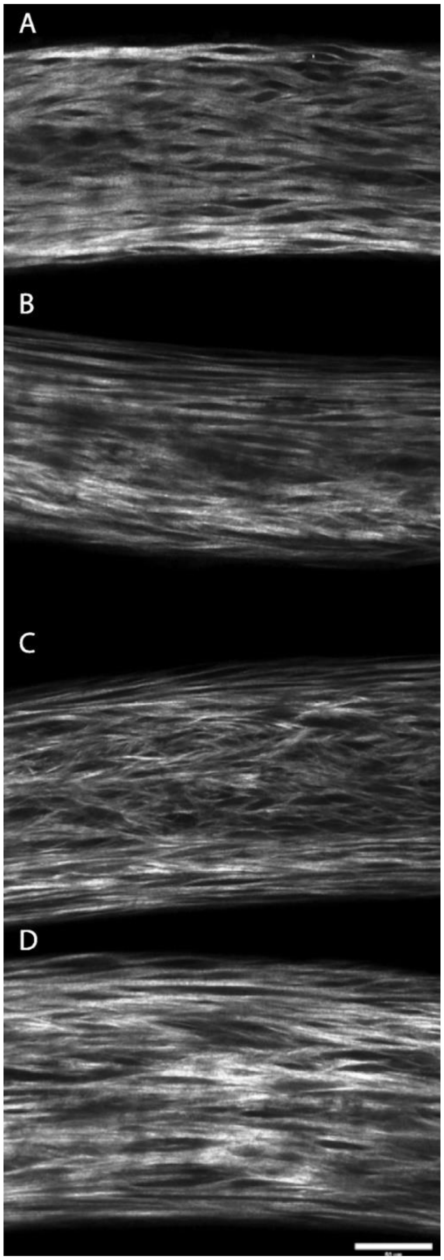

Supplemental Figure 9. TGF-β1 treated tissues show a non-monotonic response in mechanical properties by 21 but no apparent differences in SHG collagen architecture. Day 21 tissues treated with 0 (A), 0.4 (B), 2.0 (C), and 10 ng/mL (D) TGF-β1 did not have apparent differences in SHG at day 21 suggesting alternate changes in cells or ECM such as crosslinking that may be responsible for the non-monotonic response in tissue mechanics. Scale bar = 50 μm.

Supplemental Videos

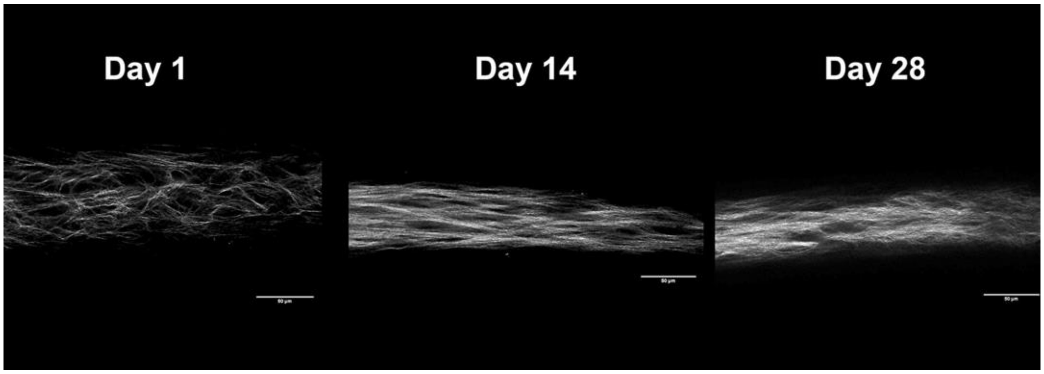

Supplemental Video 1. Tissues form a 3D network of fibrillar collagen. Tissue rings seeded with 3 x 10^5 cells were cultured in 50:50 media and imaged with SHG microscopy at days 1, 14 and 28 to reveal the 3D organization of the fibrillar collagen architecture. Multiphoton SHG isometric z-stacks show a clear shift in the density and organization of fibrillar collagen in 3D from day 1 to day 14 with more subtle changes from day 14 to day 28. This corresponds to changes in the total collagen content assessed biochemically as well as the mechanical properties of tissue constructs. This technique provides a powerful tool to map structure to function by non-destructively tracking changes in collagen architecture.

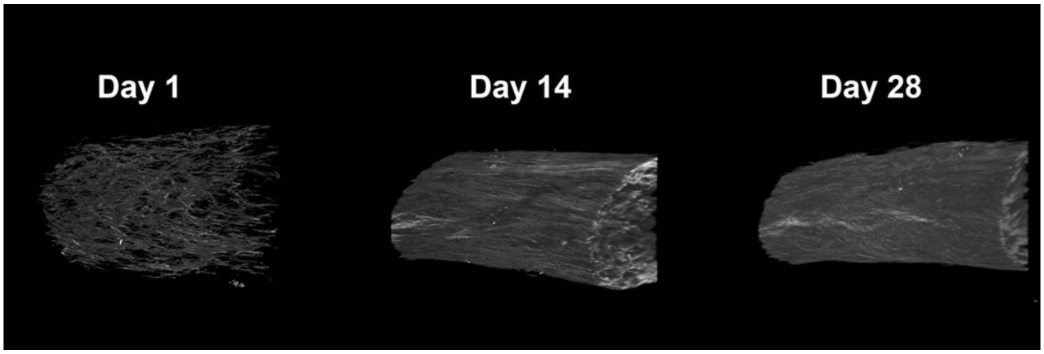

Supplemental Video 2. Tissues form a 3D network of fibrillar collagen. Tissue rings seeded with 3 x 10^5 cells were cultured in 50:50 media and imaged with SHG microscopy at days 1, 14 and 28. 3D reconstruction of the SHG mages shows changes in the volume density of collagen over time characterized by a significant increase in collagen alignment and density from day 1 to day 14.

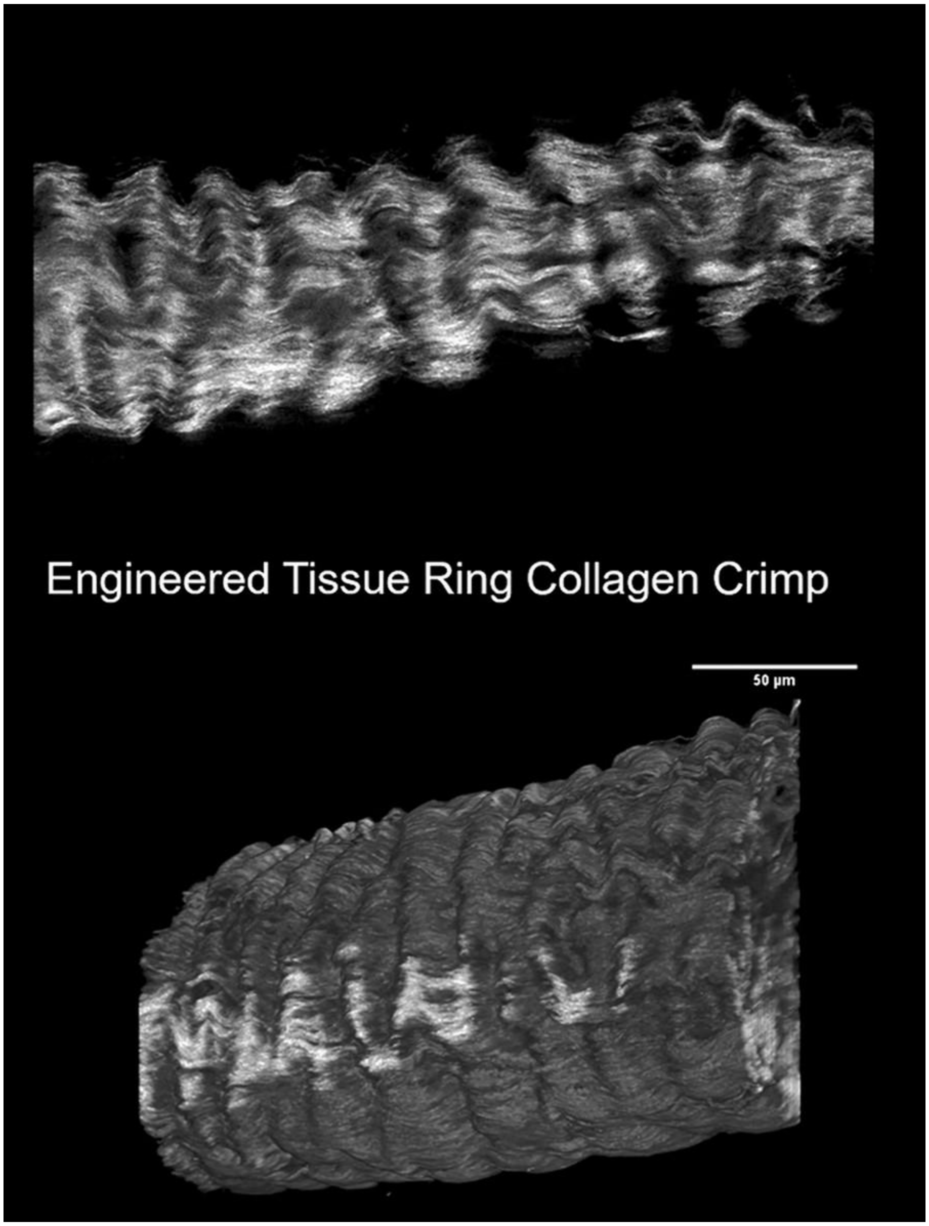

Supplemental Video 3. Ring-shaped tissues develop cell-mediated tension that results in crimping of collagen over time. Tissue rings seeded with 3 x 10^5 cells that were cultured in 50:50 media for 28 days were removed from the agarose pegs and immediately placed into fixative to capture the instantaneous relaxation of tension in the tissues. Tissues were imaged using SHG microscopy and a 3D reconstruction of the images shown. Rings exhibited collagen crimping behavior characteristic of highly-aligned, collagen-rich soft tissues like ligaments and tendons.

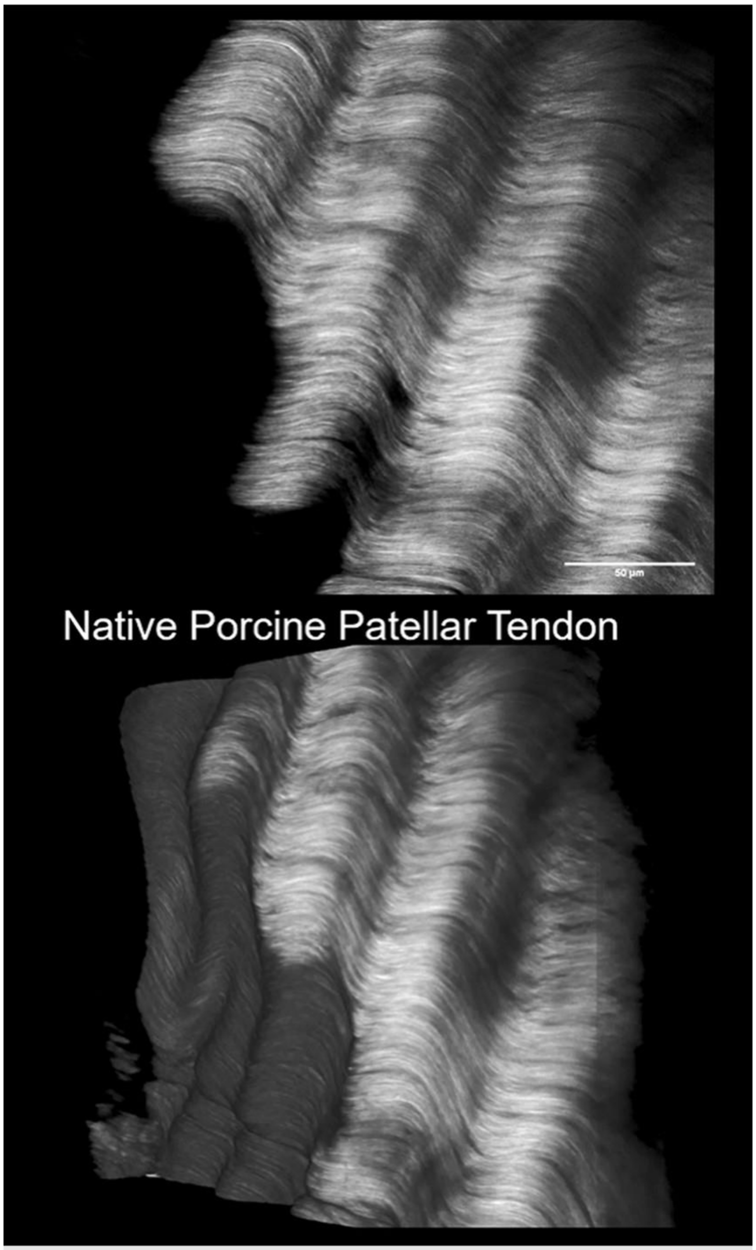

Supplemental Video 4. To compare collagen crimping behavior of our engineered ring-shape tissue constructs to native tissue, we obtained porcine patellar tendon and performed multiphoton SHG microscopy to obtain 3D information on the collagen crimp of mature native tendons. As expected, native tissues exhibited highly ordered crimping behavior with qualitative increases in wavelength and amplitude compared to engineered tissue constructs.

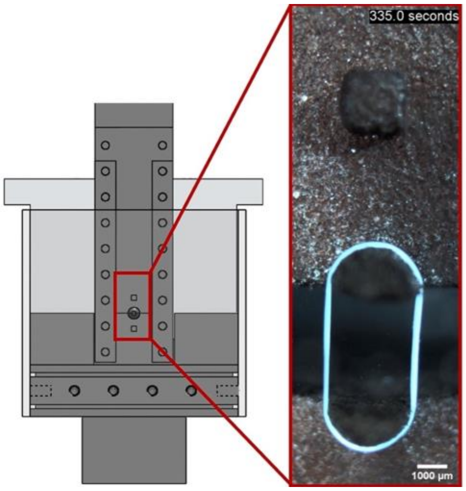

Supplemental Video 5. Video of mechanical testing of human tissue rings. To perform tensile load to failure tests on our engineered rings, we designed custom grippers to enable loading and testing of rings using an Instron 5943 equipped with a 5N load cell. We designed and fabricated a custom enclosure to enable aqueous, heated testing in PBS at 37◦C. Grippers consisted of two semicircle cylinders, each with a radius of 1.5 mm to enable the loading of 5 mm rings. Prior to testing, tissue constructs were gently removed from the agarose mold using forceps and brought to a 5 mm initial grip-to-grip distance and subsequently tested at 0.1%/s corresponding to 5 *µm* per second to approach quasi-static loading.
